# Metallic Glass/PVDF Magnetoelectric Laminates for Resonant Sensors and Actuators: A Review

**DOI:** 10.3390/s17061251

**Published:** 2017-05-31

**Authors:** Jon Gutiérrez, Andoni Lasheras, Pedro Martins, Nélson Pereira, Jose M. Barandiarán, Senentxu Lanceros-Mendez

**Affiliations:** 1 BCMaterials, Technology Park of Biscay, Building 500, 48160 Derio, Spain; manu@bcmaterials.net (J.M.B.); senentxu.lanceros@bcmaterials.net (S.L.-M.); 2 Departamento de Electricidad y Electrónica, Universidad del País Vasco UPV/EHU, P.O. Box 644, 48080 Bilbao, Spain; andoni.lasheras@ehu.es; 3 Centro/Departamento de Física, Universidade do Minho, 4710-057 Braga, Portugal; pmartins@fisica.uminho.pt (P.M.); nmmsp.18@gmail.com (N.P.); 4 IKERBASQUE, Basque Foundation for Science, 48013 Bilbao, Spain

**Keywords:** magnetoelectrics, magnetoelectric heterostructures, magnetoelectric sensors

## Abstract

Among magnetoelectric (ME) heterostructures, ME laminates of the type Metglas-like/PVDF (magnetostrictive+piezoelectric constituents) have shown the highest induced ME voltages, usually detected at the magnetoelastic resonance of the magnetostrictive constituent. This ME coupling happens because of the high cross-correlation coupling between magnetostrictive and piezoelectric material, and is usually associated with a promising application scenario for sensors or actuators. In this work we detail the basis of the operation of such devices, as well as some arising questions (as size effects) concerning their best performance. Also, some examples of their use as very sensitive magnetic fields sensors or innovative energy harvesting devices will be reviewed. At the end, the challenges, future perspectives and technical difficulties that will determine the success of ME composites for sensor applications are discussed.

## 1. Introduction

The magnetoelectric (ME) effect is defined as the electrical field (or voltage) induced under the application of a magnetic field (direct ME effect), or vice versa, as the magnetic induction under the application of an electrical field (inverse ME effect). In 1894 the intrinsic ME effect was theoretically predicted by Pierre Curie, and was experimentally first observed nearly 60 years ago in single-[1] and poly-crystals [2] of single-phased materials, but it turn out to be a weak effect observed only at low temperatures. To make the magnitude of this effect useful for applications, a good alternative was to convert the known ME materials to composite systems in which one of the constituent was purely magnetic/magnetostrictive and the other one, purely piezoelectric. These composite heterostructures were of the type “particulate” and “laminate” ones. The first particulate composites were made of magnetostrictive ferrites and piezoelectric Pb(Zr,Ti)O_3_ (or PZT) and gave values of the induced magnetoelectric voltages up to 0.4 V/cm·Oe [3,4]. However, these new hybrid systems exhibited some problems as chemical reactions between the starting materials during the sintering process, or mechanical defects that limited the mechanical coupling between the particles of the constituents [5].

These problems were overcome in 2001 by using laminate composites, with layers of magnetostrictive and piezoelectric phases epoxied together. Ryu et al. [6] reported a ME voltage of 4.68 V/cm·Oe in a structure consisting in a disc of PZT sandwiched between two discs of Terfenol-D. Nevertheless, despite the good performance of this type of magnetoelectric laminates some problems still remained, such as brittleness, low permeability and the high applied magnetic fields needed to achieve the maximum ME effect for the magnetostrictive phase (mostly Terfenol-D), and also brittleness and high resistance of the piezoelectric component (mostly PZT) to reduce eddy current losses of the composite.

New combinations of magnetostrictive/piezoelectric layers were needed; in response, by using high permeability magnetostrictive materials such as iron-based Metglas alloys epoxied to poly(vinylidene fluoride)(PVDF) piezoelectric polymer [7], signals as high as 7.2 V/cm·Oe at low (sub-resonant) frequency and 310 V/cm·Oe at the electromechanical resonance of the composite, were obtained. This electromechanical resonance takes place when a mechanical resonant response is excited through the magnetostrictive effect of the magnetic constituent of the laminate, or what is equivalent at its corresponding magnetoelastic resonance (MER) frequency.

To account for such results, we have to analyze the ME effect magnitude, that is usually defined as the product between the piezomagnetic and piezoelectric effects [8]:

(1) $$\alpha_{ME}=\frac{dE}{dH}=k_{c}\left(\frac{\delta \lambda }{\delta H}\right)\left(\frac{\delta E}{\delta \lambda }\right)$$

for laminates with unconstrained longitudinal vibration. In this equation *δλ/δH* is the piezomagnetic coefficient of the magnetic element, *δE/δλ* is the piezoelectric constant of the dielectric one and *k_c_* is the coupling constant (arisen from bonding conditions) between both constituents. So, even if magnetostriction of Terfenol-D, *λ* > 1000 ppm, is much higher than the magnetostriction of a Metglas-like amorphous magnetic material with *λ* ≈ 40 ppm in the best cases, the quantity that drives the ME is actually the piezomagnetic coefficient of the magnetic constituent, $\delta \lambda /\delta H=d_{33}^{m}$; while for Terfenol-D the maximum value of $d_{33}^{m}$ *=* 1.2 × 10^−6^/Oe occurs at about 500 Oe applied external magnetic field [9], for commercial or home-made Metglas-like amorphous materials this maximum $d_{33}^{m}$ value is almost equal, but it can be achieved at an applied field of only a few Oe.

Among the piezoelectric compounds some specific functional polymers such as poly(vinylidine fluoride) (PVDF) polymer and its copolymer poly(vinylidene fluoride/trifluoroethylene) P(VDF-TrFE) have been widely used and optimized as piezoelectric matrix on ME structures due to their interesting ferroelectric and piezoelectric properties [10]. They have a moderate piezoelectric coefficient of a few pC/N, but they show the advantages of being strongly flexible as well as used in film form, which makes it very useful to conform surfaces of different shape. Notwithstanding the higher ME coefficients being reported on piezoelectric ceramic-based composites, polymer-based ME composites offer more simple elaboration process, absence of brittleness and fragility, low electrical resistivity and high dielectric losses.

The mechanism underlying the ME effect in laminates is easy to understand: the magnetostrictive constituent will deform under the action of an applied external magnetic field, H. This strain will transmit to the piezoelectric material layer through elastic bonding with an epoxy between both constituents. Finally, this deformation of the piezoelectric material will give rise to an induced ME voltage through piezoelectricity.

The close collaboration among the BCMaterials Research Center, the Group of Magnetism and Magnetic Materials of the Universidad del País Vasco (UPV/EHU), both from the Basque Country, Spain, and the Electroactive Smart Materials Group from the Universidade do Minho at Braga and Guimaraes, Portugal, has given as result an intense and fruitful activity devoted to the development of Metglas-like/PVDF laminated magnetoelectric composites with high response that have been tested for high sensitivity sensors and actuators based on the previously mentioned effect. In the following, we present the relevant background on the fabrication of polymer-based ME laminates and their characterization methods and discuss the key considerations in the selection of materials and in the design of these ME devices. We summarize the latest results concerning magnetic sensors and energy harvesters based on Metglas-like/PVDF ME laminates, as well as address the mains challenges and prospects for the near future.

## 2. Experimental

### 2.1. Materials: Magnetostrictive and Piezoelectric Constituents

Concerning the magnetostrictive constituent, all metallic glasses mentioned in this work are Vitrovac 4040 (Fe_39_Ni_39_Mo_4_Si_6_B_12_) or home-made samples with nominal compositions (Fe_0.79_Co_0.21_)_75 + x_Si_15 − 1.4x_B_10 + 0.4x_ (x = 0, 3, 6) and Fe_85 − x_Co_x_B_15_ with x = 21. All these are Fe-based metallic glasses containing Fe-Co-Ni-Si-B in their composition and were prepared by the single roller quenching method in the form of long ribbons. Different pieces of the same ribbon were cut to perform their magnetic and magnetoelastic characterization. Room temperature hysteresis loops were measured by a classical induction method, obtaining so saturation magnetization (*μ_o_M_s_*) and susceptibility (*χ*) values. Magnetostriction (*λ*) was determined by using a strain gage connected to an electronic Wheatstone bridge. Any elongation arising from temperature effect was compensated by using a parallel connected free standing strain gage. From this measurement the piezomagnetic coefficient $d_{33}^{m}=\delta \lambda /\delta H$ was determined. Extensive magnetoelastic resonance measurements have been performed to determine resonant (*f_r_*) and anti-resonant (*f_a_*) frequencies and the signal amplitude at the resonance for all the studied samples, as it will be extensively explained in the following subsection. Table 1 summarizes the obtained mean magnetic parameters for all the used magnetostrictive alloy compositions [11].

Concerning the piezoelectric polymer it was used PVDF, the well-known piezoelectric polymer [10,13]. It shows moderate piezoelectric coefficients, ranging as |$d_{33}^{p}$| ≈ 24–34 pC/N and $d_{31}^{p}$ ≈ 8–22 pC/N. In addition, it has glass transition and melting temperatures about −35 °C and 171 °C, respectively, but a Curie temperature of ≈ 100 °C.

**Figure 1:**
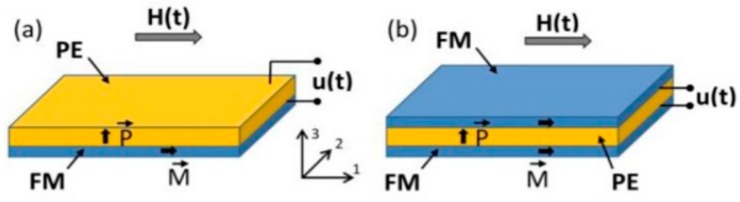
Geometry of a (**a**) bilayer MP and (**b**) three-layer sandwich-like (L-T type) MPM structures. u(t) is the alternating induced ME voltage. Figure taken with permission from [16].

Polymer-based ME laminates are typically produced by epoxing magnetostrictive layers (Metglas-like or Vitrovac; M) to commercial poled *β*-PVDF (P), following the optimized conditions presented in previous studies [14,15]. Briefly, Young’s modulus and thickness of the epoxy as well as thickness of the piezoelectric layer have to be taking into account [14]. All the laminates appearing within this work are bilayers (MP; see Figure 1a) or three-layer sandwich-like (MPM and L-T type, where L stands for magnetization in plane of magnetostrictive ribbon and T stands for transversely poled piezoelectric film; see Figure 1b) laminated composites. After the epoxing procedure between the Metglas-like ribbon and the PVDF piezoelectric layer, the hysteresis loop of the whole laminated was measured again in order to detect any change in the anisotropy of Metglas due to induced stresses arising from this bonding process.

### 2.2. From the Magnetoelastic Resonance to the Induced Magnetoelectric Effect

The Fe-based metallic glasses used in the fabricated ME laminates show an excellent coupling between magnetic and elastic properties, and as well as an applied external magnetic field causes magnetostrictive deformation of the magnetic material, the inverse effect also happens. That is, any application of a mechanical stress causing a deformation in the ferromagnetic material, will cause a change in its magnetic state. This is the so called magnetoelastic or Villari effect. A direct consequence of such magnetoelastic coupling is the dependence of elastic constants of magnetostrictive materials with applied external magnetic field, which in the case of magnetostrictive amorphous long ribbons translates to a clear dependence of longitudinal Young’s modulus with *H* or Δ*E* effect.

It turns out this is easy to measure since magnetization changes can be detected inductively; thus, if longitudinal deformations of the metallic glass ribbon piece are excited through magnetostriction, the elastic sound wave induced in the sample will be accompanied by a magnetization one, giving rise to a magnetoelastic wave (a detailed mathematical formalism can be found in [17]).

Driving the induced elastic wave adequately by changing the frequency of the applied external magnetic field, the detected magnetoelastic wave will become stationary and will enter to a resonant state at a resonant frequency *f_r_*. It is possible to use a home-mounted magnetoelastic resonance detection apparatus that automatically changes the external applied magnetic field (or bias) *H_dc_* and the value of the frequency of the *H_ac_* magnetic excitation in order to drive the sample to its magnetoelastic resonance at a given *H_dc_*, and stores the correspondent frequencies for the maximum (or resonant, *f_r_*) and minimum (or anti-resonant, *f_a_*) induced signals, together with the signal amplitude at the resonance [18,19]. These measured frequencies and mainly the resonant (*f_r_*) one will vary with the bias field *H*, and so it will do the Young’s modulus determined as:

(2) $$E(H)={\left[2Lf_{r}(H)\right]}^{2}\rho$$

where *L* and *ρ* are the length and density of the sample. This field-dependence of this elastic modulus is known as Δ*E* effect (Δ*E* = 1 − *E(H)/E_S_*, *E_S_* being the Young’s modulus measured at magnetic saturation). Other useful magnetoelastic parameters that can be determined from these measurements are the magnetomechanical coupling coefficient (*k* = (π^2^/8)(1 − (*f_r_*/*f_a_*)^2^)) and quality factor of the resonance (*Q* = *f_r_/*Δ*f*), all quantities being function of the applied external magnetic field.

**Figure 2:**
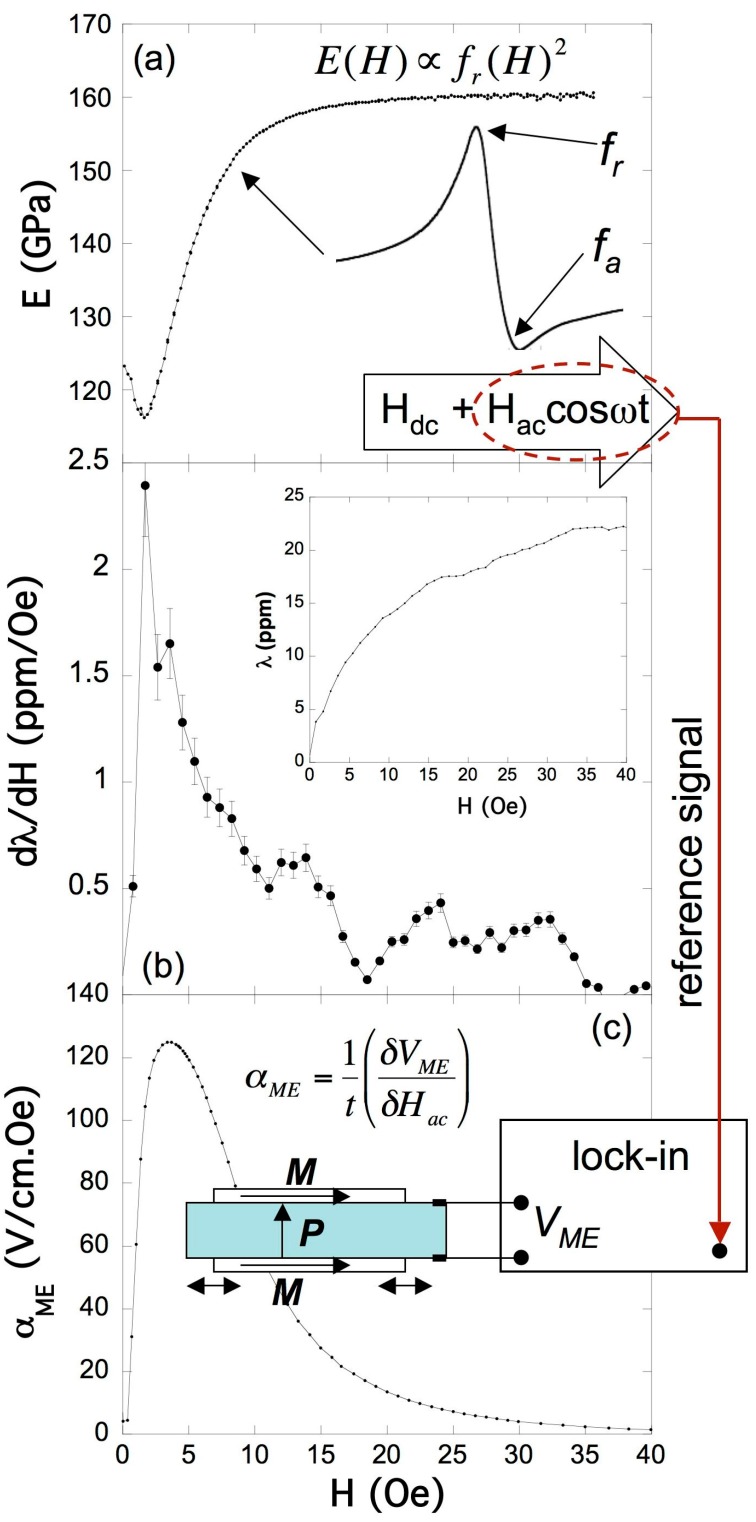
(**a**) Magnetoelastic resonance; (**b**) piezomagnetic coefficient or magnetostriction field derivative (the inset shows the magnetostriction curve) measurements performed on a long ribbon of as-cast Fe_64_Co_17_Si_6.6_B_12.4_ amorphous magnetostrictive material; (**c**) ME measurements performed in a three-layer L-T sandwich configuration: the same previous magnetostrictive ribbon is longitudinally magnetized while the piezoelectric polymer (PVDF) is transversely poled.

Figure 2a shows an example of such magnetoelastic resonance measurements performed on a 30 mm (length) × 1.8 mm (width) × 30 mm (thickness) long ribbon of as-cast Fe_64_Co_17_Si_6.6_B_12.4_ amorphous magnetostrictive material. The maximum value of the Δ*E* effect occurs at low fields where magnetostriction has not achieved its maximum value (see inset in Figure 2b), but yes its derivative or piezomagnetic coefficient *δλ/δH* (Figure 2b).

Taking into account Equation (1), it immediately arises the fact that maximum magnetoelectric induced voltage will be found at the maximum value of *δλ/δH*, usually at applied *H_dc_* fields close to the corresponding maximum value of the Δ*E* effect (*E(H)* minimum value) and always at the electromechanical resonance of the laminate composites.

Thus, in order to measure the ME effect magnitude one only needs to slightly modify the former magnetoelastic resonance apparatus: coaxial solenoids with the ME laminate in its centre apply a net magnetic field *H*(*t*) = *H_dc_* + *H_ac_*cos*ωt* (*H_ac_* << *H_dc_*) on it. It is necessary first to determine the static field *H_dc_* needed for maximum amplitude of the magnetoelastic resonance.

Under a *H_ac_* magnetic excitation applied along the length axis, the magnetostrictive ribbons will elongate and shrink along that direction. This will make the piezoelectric film of PVDF to undergo an *ac* longitudinal strain, inducing a dielectric polarization change in its transverse direction that is accurately measured as a magnetoelectric voltage *V_ME_* by using a lock-in amplifier (Figure 2c). From this voltage the magnetoelectric coefficient *α_ME_* can be directly obtained as [6]:

(3) $$\alpha_{ME}=\frac{dE}{dH}=\frac{1}{t}\left(\frac{\delta V_{ME}}{\delta H_{ac}}\right)$$

A clear advantage of this measurement method is that the same experimental set-up allows us to determine simultaneously: (a) the ME response dependence as the bias field *H_dc_* changes and (b), the magnetoelectric voltage dependence against the applied *H_ac_* magnetic excitation, the so called *sensitivity* of the ME laminate.

## 3. Some applications of Magnetoelectric Laminates

### 3.1. Magnetic Field Sensor

From Figure 2a,c it is clearly inferred that linearity in the response is maximum for applied external magnetic fields within the range from 0 to the corresponding one for the minimum of the Δ*E* effect (or maximum of the induced ME signal), that turns out to be usually of a few Oe of magnitude.

**Figure 3:**
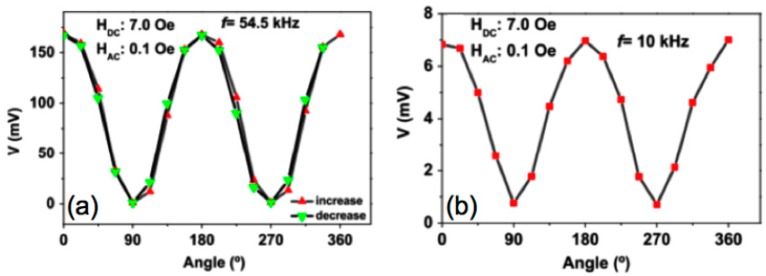
ME voltage value variation with the 0°–360° angle range at: (**a**) resonance frequency and (**b**) non-resonance frequency. Figure taken with permission from [20].

ME devices with their capacity accurate measure of magnetic fields can solve some problems of traditional magnetic sensors such as high signal noise, the inability to measure magnetic fields at precise locations and the cross-sensitivity between measurement axes due to angular errors. Meeting the nowadays increasing demands for vector magnetometers the anisotropic ME voltage response on a Fe_61.6_Co_16.4_Si_10.8_B_11.2_/PVDF/Fe_61.6_Co_16.4_Si_10.8_B_11.2_ laminate has been utilized for the development of a magnetic field sensor (2 cm × 1 cm size) capable to sense the magnitude and direction of both AC and DC magnetic fields (see Figure 3).

The linearity (92% and 99% for the DC sensor and for the AC sensor respectively), accuracy (99% for both AC and DC sensors), and reproducibility (99% for both AC and DC sensors) proved the appropriateness of the sensor for device applications. Additionally, the sensitivity of the laminate anisotropic magnetic field sensor (15 and 1400 mV/Oe for the DC and AC fields respectively) were the highest stated in the literature for polymer-based ME materials. Such performance, combined with the versatility, flexibility, low cost, light weight, and low temperature production are enormous advantages of developed ME materials for utilization in magnetic sensor device applications [20].

Once key parameters such as accuracy, sensitivity, linearity, resolution and hysteresis have been only unclearly discussed in the literature Reis et al. reported on those performance characteristics on a Metglas/PVDF/Metglas ME laminate [21].

**Figure 4:**
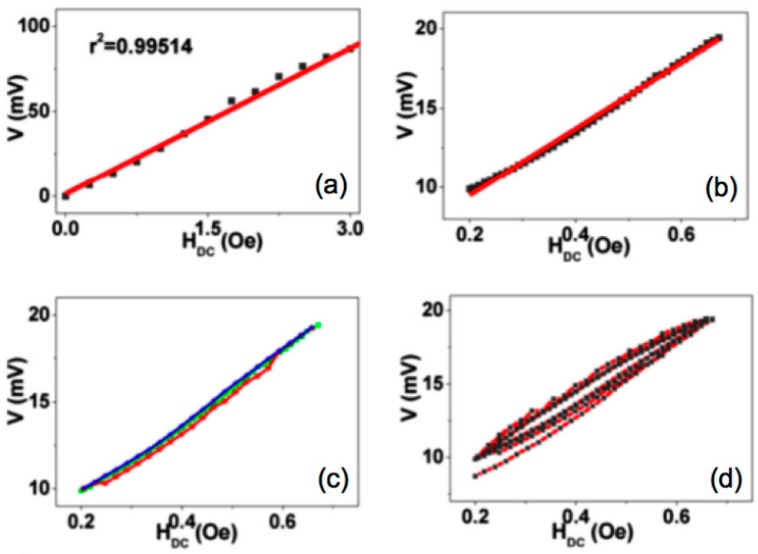
DC magnetic field sensor characterization: (**a**) linearity, (**b**) resolution and sensitivity (**c**) accuracy and (**d**) hysteresis. Figure taken with permission from [21].

The sensitivity and resolution determined for the AC magnetic field sensor (992 mV/Oe and 0.3 µOe) and DC magnetic field sensor (30 mV/Oe and 8 µOe) were positively comparable with the most sensitive polymer-based ME sensors (see Figure 4). Furthermore, the correlation coefficient, accuracy and linearity obtained values were 0.995, 99.4% and 95.9% for the DC magnetic field sensor and 0.9998, 99.2% and 99.4% and for the ME AC magnetic field sensor. Consequently, the ME materials developed in such work can be used for pioneering AC/DC magnetic field sensors device applications [21].

**Figure 5:**
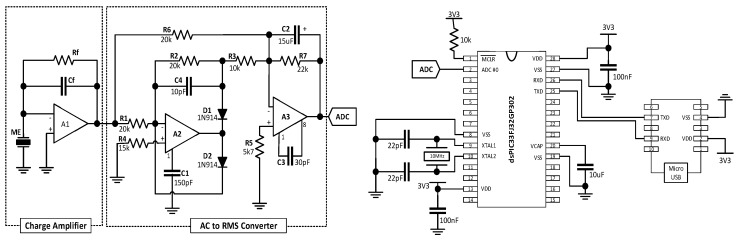
(**left**) ME signal modulation circuit with two distinct stages: charge amplification resulting in an AC voltage output signal and AC-to-DC voltage converter. (**right**) Analog-to-digital converter circuit to which the previous analogue circuit was connected.

Taking advantage of the same materials and incorporating a charge amplifier, an AC-RMS converter and a microcontroller on chip peripheric analogue to digital converter (ADC) it was developed of a DC magnetic field sensor with readout electronics (see Figure 5, adapted from [22]).

The ME voltage output was not distorted, the linearity was preserved and the ME voltage response was found to increase to 3.3 V (α_33_ = 1000 V/cm·Oe) with the introduction of the electronic components. The sensing device, including the readout electronics, revealed a maximum drift of 0.12 Oe with an average total drift of 0.04 Oe, a 70 nT resolution and a sensitivity of 1.5 V/Oe. Such performance was for the first time reported on a polymer-based ME device and was favorably comparable with a reference Hall sensor that showed a maximum drift of 0.07 Oe and an average error of 0.16 Oe, 5 V/T sensitivity and 2 µT resolution.

This device performance associated to the precise H_DC_ fields measurement mark this polymer-based device as very attractive for device applications such as digital compasses, Earth magnetic field sensing, magnetic field anomaly detectors and navigation, among others. Nevertheless the successful implementation of ME sensors is intimately connected with the production of those materials through advanced assembly techniques such as printing, which may induce optimized properties and consent better technology transfer to the industry. Additionally costume polymer-based ME sensors implies two magnetic inputs: a DC magnetic field and AC magnetic field. Both types of magnetic field can be detected by inputting the other, resulting in a magnetic sensor able to sense DC or AC magnetic fields. This required complementary magnetic field makes more complex the design of the magnetic sensing devices. In his way, the development of innovative self-biased ME sensors that can be used as passive ME magnetic field sensors in the absence of the DC magnetic field is a huge milestone in this dynamic research field.

### 3.2. Energy Harvesters

The use of polymer-based ME laminated composites as energy harvesting devices has increased in the last years [23,24]. These type of harvesters are usually fabricated with ferromagnetic metallic glasses as the magnetostrictive constituent and the polyvinylidenefluoride (PVDF), not only because of their high ME response, but also due to their excellent mechanical properties and low cost of production [25]. Also, the performance of such energy harvesting device is proportional to the induced ME voltage, and so its best performance will be achieved at the DC magnetic field needed for the maximum ME coupling. The low DC magnetic field required for the polymer based ME laminated composites greatly simplifies the implementation of the ME laminated composites, in comparison with other ME energy harvesting devices, as it will be discussed in the following.

Due to the ME effect measurement process, to rectify the AC signal coming from the ME laminates and convert it into a DC one turns out to be the first important step to be solved. There are in the literature many circuits for this purpose that can be used and applied to energy harvesters. In fact, the output power showed by the laminates will depend on the characteristics of the circuit used, a key factor which makes necessary its optimization. Four are the most common energy harvesting circuits: a full-wave bridge voltage rectifier, two Cockcroft-Walton voltage multipliers with one and two stages and a three stages Dickson voltage multiplier.

**Figure 6:**
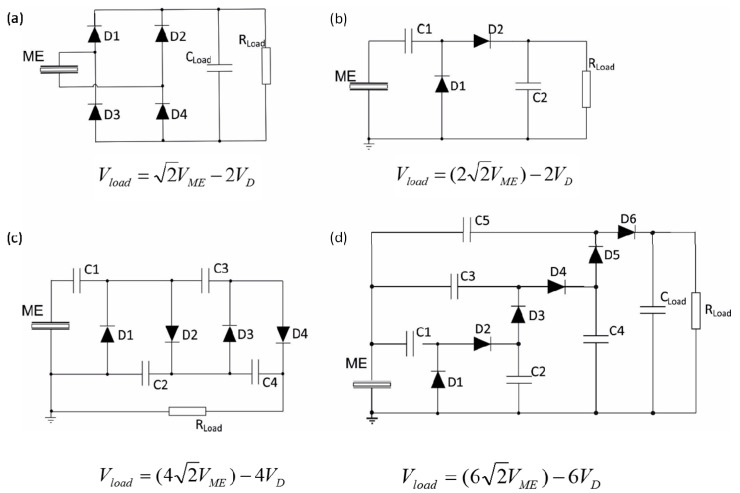
Schematic representation of a full-wave bridge voltage rectifier (**a**); one-stage Cockcroft-Walton voltage multiplier (**b**); two-stages Cockcroft-Walton voltage multiplier (**c**) and three stages Dickson voltage multiplier (**d**). *V_ME_* represents the induced voltage in the ME laminate, *V_D_* represents forward voltage drop across each diode and *V_Load_* measured voltage at the load resistance. Figure adapted from reference [11], with permission of the author.

The full-wave bridge voltage rectifier circuit is widely used in energy harvesting systems that converts AC voltage to DC voltage [26,27]. The main advantages of this circuit are the low energy loss, low complexity and high efficiency. This circuit consists of four Schottky diodes (see Figure 6a) which convert the AC output voltage of the ME laminate into a DC one through the two half cycles (positive and negative).

The voltage multipliers efficiently convert the AC signal into a DC one and simultaneously increase the output voltage [28]. The Cockcroft-Walton circuit is a half-wave rectifier constituted by n stages, each stage formed by two diodes and two capacitors (see Figure 6b,c).

The one-stage Cockcroft-Walton voltage multiplier (see Figure 6b) consists on a clamper constituted by the capacitor C1 and the diode D1 and a peak detector constituted by the capacitor C2 and the diode D2. The clamper signal is measured in the diode D1 and corresponds to the wave input shifted from the negative peak to zero. The peak detector assigns a DC voltage with approximately twice the input peak voltage value. The two-stage Cockcroft-Walton voltage multiplier (see Figure 6c) has a similar behavior than the previous one but the input signal is increased four times by adding another multiplier level.

The Dickson voltage multiplier circuit is also a half-wave rectifier, which can be constructed with n stages, being each one formed by two diodes and two capacitors. The Dickson multiplier showed in Figure 6d, is a three-stage circuit based on the original Dickson charge pump, a DC-DC converter where the original DC input is shunted to the ground level and the logic control is replaced by the AC input signal to be harvested [29]. Extensive information about all circuits together with component values can be found in [27].

These four different circuits were tested using a 3 cm long Fe_64_Co_17_Si_6.6_B_12.4_/PVDF/ Fe_64_Co_17_Si_6.6_B_12.4_ three-layered ME laminate, working at the L-T configuration and at its magnetoelastic resonance frequency, measured to be 41.6 kHz. The total cross section of the ME laminate is 2.5 × 0.078 mm^2^ and the metallic glasses have been previously annealed at 300 °C for 10 min in order to release the internal stresses arisen in their fabrication.

The obtained output ME voltage was continuously monitored by a Hewlett Packard 54603 oscilloscope and subsequently the corresponding output electric power was obtained when varying the load resistance from 1 kΩ to 1 MΩ.

**Figure 7:**
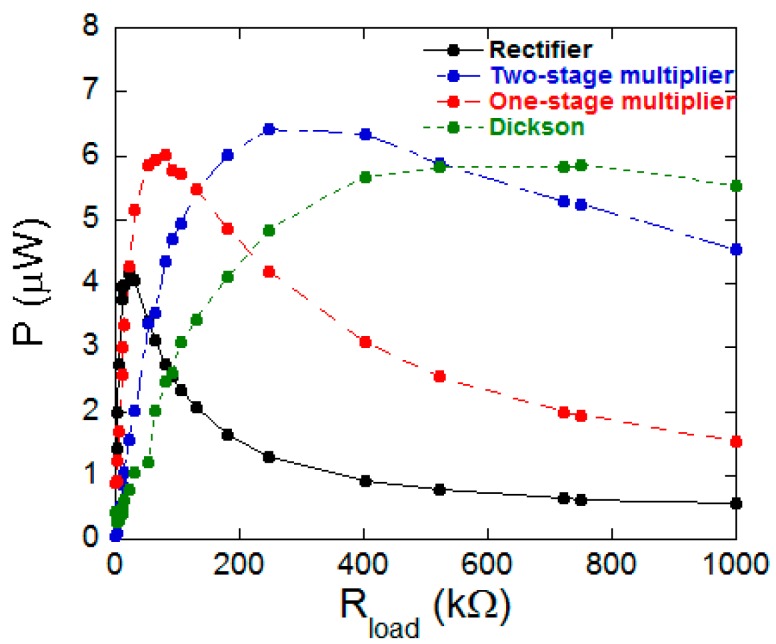
The output electric power as a function of the load resistance for all the studied energy harvesting circuits.

As it can be observed in Figure 7, the maximum output electric power (6.4 µW) is achieved for the two-stage multiplier circuit when a load resistance of 250 kΩ is used. Considering the total volume of the laminate, the corresponding power density value has been estimated to be 1.5 mW/cm^3^. It is remarkable that this obtained maximum (magnetoelectric) power generated value is comparable to some previously reported power densities for laminates containing PZT and PVDF (see Table 2) as piezoelectric constituents.

Therefore, the used two-stage multiplier circuit working with a high performance ME device (3 cm × 5 mm size) could act as simple and low cost ME effect based energy harvester with good output electric power response. It has to highlight that the obtained output value is within the ultra- low-power consumption devices suitable for biomedical wireless communications systems [34], among others.

## 4. Size Effects on the Induced Magnetoelectric Signal

When dealing with applications, not only the good bonding between the piezoelectric and magnetostrictive constituents plays an important role, but also other factors as size and relative geometry of the components are of great importance [35]. In the following we will focus on these aspects that, as it will be shown, strongly affects the magnetoelectric laminates performance.

### 4.1. Size Effects

**Figure 8:**
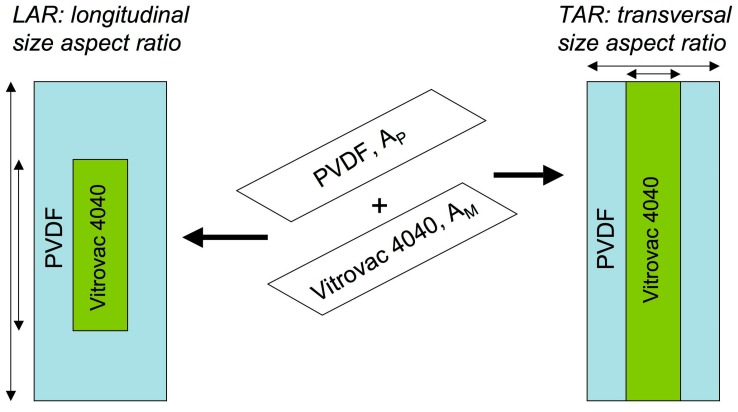
Graphical definition of the longitudinal size aspect (LAR) ratio and transversal size aspect (TAR) ratio between the PVDF and Vitrovac layers.

Silva et al. was the first report directed to study the influence of the relative size of the magnetostrictive and piezoelectric elements on the ME response [36]. To do this, rectangular pieces of magnetostrictive Vitrovac 4040 (Fe_39_Ni_39_Mo_4_Si_6_B_12_) and poled *β*-PVDF where cut in several different widths and lengths. In this way, ME laminates with different longitudinal size aspect (LAR) ratio and different transversal size aspect (TAR) ratio between the PVDF and Vitrovac layers were fabricated (see Figure 8).

The obtained results clearly indicated that the ME induced voltage increases with decreasing the LAR ratio value, while laminates with the lowest TAR resulted in better ME performance when compared with higher TAR ratio values. Both those aspect rations, even if being different, further demonstrated a clear correlation with the quantity *Area_PVDF_/Area_Vitrovac_* = *A_P_/A_M_*: the ME response was always optimized for values *A_P_/A_M_* ≈ 1.

Following this line of work, Laheras et al. proceeded to deeply investigate the case of ME laminates with equal sizes of piezoelectric and magnetostrictive constituents (value *A_P_/A_M_* ≈ 1) and different length values of the laminates. To do this, they were fabricated 3, 2, 1 and 0.5 cm long, three-layered Longitudinal-Transverse (L-T) structures of PVDF located between two magnetostrictive ribbons of composition Fe_61.6_Co_16.4_Si_10.8_B_11.2_, and the ME response was studied [37].

The first observation, as expected, is that the shorter the laminate, the higher the working frequency of the device, since this frequency matches with the magnetoelastic resonance (MER) frequency of the magnetostrictive constituent (49.1 kHz, 70.8 kHz, 165.5 kHz and 303.7 kHz for the 3, 2, 1 and 0.5 cm long laminates, respectively). Concerning the loss of the ME signal arising from the increase of this working frequency, it is originated by both the magnetostrictive and piezoelectric constituents, but has been quantified to be small enough to be neglected.

As a direct consequence, the observed loss of the induced ME signal must be inherent to the decrease of the size of the laminate. In fact and for the magnetostrictive constituent, demagnetizing effects cannot be neglected, since they become stronger as the length of the magnetostrictive ribbon used decreases. Even if demagnetizing factors for three-layer laminated composites have been already theoretically quantified [38], when the thickness of the piezoelectric layer is very thin, it is possible to calculate the demagnetizing factor of the whole laminated composite by using the following expression:

(4) $$N_{lam}\approx \frac{1}{2}(N_{t}+N_{2t})\approx \frac{3}{2}N_{t}$$

where *N_t_* and *N_2t_* are the experimentally determined demagnetizing factors of prism-shaped ribbons of thicknesses *t* and *2t*, respectively [39]. For very thin ribbons (*t* → 0), *N_2t_* ≈ 2*N_t_* [39]. A good estimation to the experimental value of *N_t_* was given by Clark et al. [40] by using the expression *H_i_ = H_e_ − N_t_M_S_*, being *H_i_* the intrinsic anisotropy field of the ribbon (obtained from the hysteresis loop of a very long ribbon) and *H_e_* the effective anisotropy field. This last, in short magnetostrictive ribbons, is given by the bias field corresponding to the maximum amplitude of the ribbon magnetoelastic resonance (or maximum induced magnetoelectric effect).

The *N_lam_* value thus obtained has demonstrated to be a good approximation to the real demagnetizing factor for the layered ME composites. Now and from the estimated demagnetizing factors for the different lengths laminates, it is possible to quantify the loss in the ME signal due to the demagnetizing fields, through the definition of the reduction factor (*RF*) corresponding to each laminate [40,41]:

(5) $$RF=\frac{1}{1+N_{lam}\chi }=\frac{\alpha_{ME}\left(N_{lam}\right)}{\alpha_{ME}\left(0\right)}$$

where *N_lam_* is the demagnetizing factor of the laminate, *χ* is the intrinsic magnetic susceptibility of the ferromagnetic alloy and *α_ME_*(0) is the intrinsic ME coefficient (that is, values corresponding to a very long, infinite ribbon and laminate, respectively, not affected by the demagnetizing fields, *N_lam_* = 0) and *α_ME_*(*N_lam_*) is the extrinsic or experimentally measured ME coefficient. This reduction factor gives the ratio of the ME voltage that is useful respect to the intrinsic ME coefficient, under consideration of demagnetizing fields. Notice that the ME intrinsic value *α_ME_*(0) represents the maximum induced ME voltage that can be extracted from the laminates, independently of their length.

**Figure 9:**
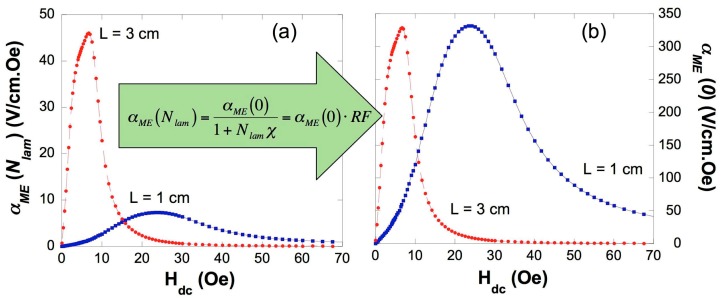
(**a**) Measured or effective ME coefficient of the L = 3 cm and 1 cm length laminates; (**b**) the corresponding intrinsic ones, both represented as a function of the applied magnetic field.

Thus, all estimated intrinsic values should be the same for the different length ME laminates, as can be directly seen in Figure 9. Nevertheless the goodness of the estimation made for the ME intrinsic value giving about 325 V/cm·Oe, it has also to be pointed that a simple calculation for the reduction factor *RF* gives for the 3 cm long laminate losses of about an 87% of the expected total ME voltage, and this loss value increases as reducing the length of the laminates, reaching the 99% for the 0.5 cm long one.

### 4.2. Quick and Direct Comparison of the Performance of Different ME Laminates

In many cases and in front of ME laminates different with different constituents, one has to afford the task to determine quickly which one is going to offer the best performance at working. When dealing with different devices of the metallic glass/PVDF/metallic glass type (that is with different magnetostrictive constituent but the same PVDF piezoelectric constituent, and also different lengths) in order to have a quick and first idea about how well each ME laminate will work, we can use the so called figure of merit, FM, that characterize the magnetic performance of the magnetostrictive material in such ME devices. This figure of merit is defined as follows [42]:

(6) $$FM=\lambda_{s}\chi$$

Thus, taking into account the previous values of the measured susceptibility and magnetostriction, it is possible to estimate the FM value for each used magnetostrictive ribbon, as summarized in Table 3.

**Figure 10:**
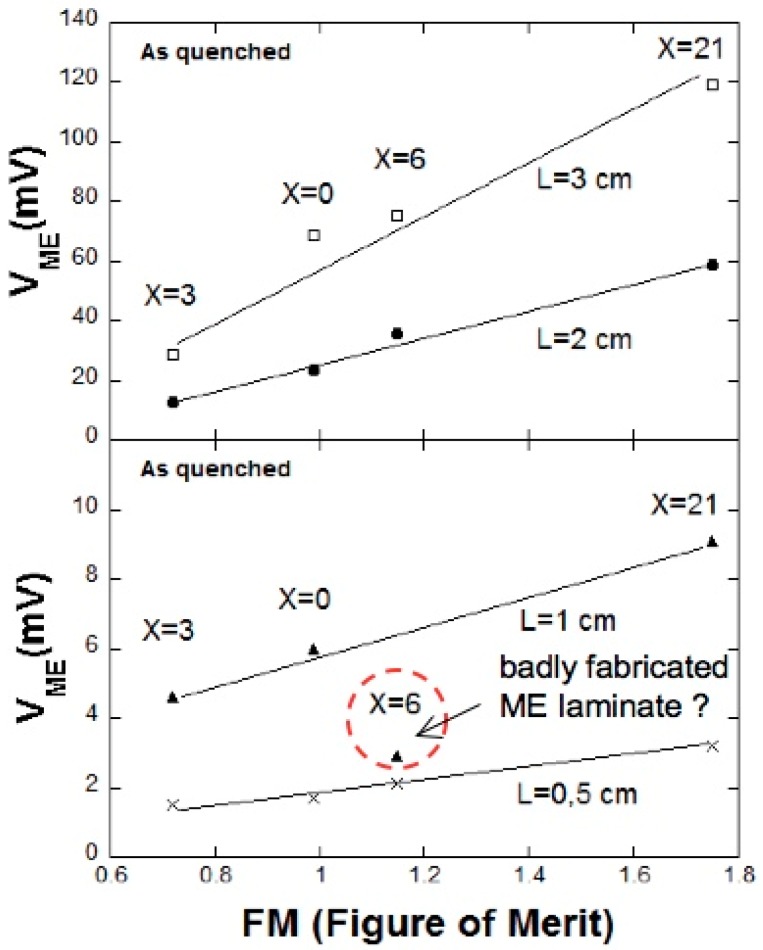
Maximum ME voltages of laminates fabricated with different magnetostrictive constituents and also different lengths, as a function of the figure of merit of each metallic glass. Figure modified from reference [11], with permission of the author.

Therefore and from all these values we can expect to get better results for ME measurements for the laminates corresponding to the X = 0/PVDF system (ribbons in as-quenched state) than for the X = 3/PVDF, despite the higher value of the magnetostriction of this last one. Nevertheless, the X = 21/PVDF laminate is expected to show the highest ME response, due to its highest magnetostriction value. To confirm this, we can draw the obtained maximum ME voltages for all the laminates fabricated with magnetostrictive ribbons, versus their corresponding (calculated) FM values (see Figure 10).

As directly observed, a good linear dependence is obtained which supports also the goodness of the definition given for the *FM* parameter. Only the 1 cm long X = 6/PVDF laminate fails to show this behavior. This disagreement can be attributed to a poor fabrication of the laminate, most probably due to a bad bonding between magnetostrictive and piezoelectric layers.

## 5. Futures Perspectives: ME Laminates for High Temperature Applications

The future tasks that ME laminates will have to address point towards miniaturization and related problems (as previously discussed) and also about working points at high temperatures. Few works have been reported so far concerning the ME effect at high temperatures (see for example [43,44]) and in most of them piezoelectric ceramic materials as PZT is often used. Most interesting and innovative studies have been mostly carried out by using PVDF (or its copolymer P(VDF-TrFE) as piezoelectric constituent. It is important to notice that PVDF shows the *α*-relaxation around 60 °C and melts ≈ 160 °C [45], limiting its use in high temperature ME laminates.

**Figure 11:**
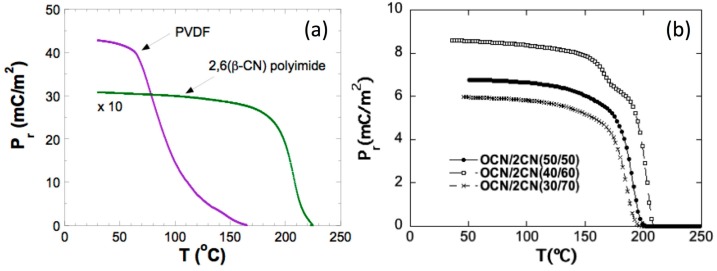
(**a**) Measured remnant polarization as a function of temperature for commercial PVDF piezoelectric polymer and the 2,6(*β*-CN)APB/ODPA (poly 2,6) polyimide; (**b**) The remnant polarization of three copolyimides as a function of temperature. Reprinted from [53] with permission of the authors. Copyright 2013 IEEE.

Nowadays there is a great interest in synthesizing new piezoelectric polymers for high-temperature applications. Among these new classes of piezopolymers, polyimides have received a lot of interest lately due to their excellent thermal, mechanical and dielectric properties [46,47,48]. Although investigations in piezoelectric polyimides have only begun recently piezoelectric polyimides have a promising future due to their interesting properties, which are suitable for many applications. Park et al. [49,50] have synthesized a polyimide, (*β*-CN)APB/ODPA, containing a single cyano dipolar group (-CN) in the repetitive unit and have shown that polyimides still maintain their piezoelectric properties at higher temperatures than commercial piezopolymers. Detailed information about synthesis, thermal characterization and electric polarization processes can be found in [48,51,52]. We only will remark briefly that its main parameters are a glass transition temperature of T_g_ ≈ 200 °C and a degradation temperature of T_d_ ≈ 510 °C, temperatures that make these polyimides suitable for high temperature purposes (see Figure 11a)). In order to combine the best mechanical and piezoelectric response in the same polymer, copolyimides can be synthesized [53] (see Figure 11b). They are usually obtained by reaction between the dianhydride ODPA and a 50% mol mixture of two aromatic diamines, namely 1,3-bis-2-cyano-3-(3-aminophenoxy)- phenoxybenzene (diamine 2CN, good piezoelectric behavior) and 1,3-bis(3-aminophenoxy)benzene (diamine 0CN, good mechanical behavior) in a two-step reaction. Extensive information about the synthesis, mechanical and dielectric and properties of these copolyimides can be found in [54,55]. Several test were performed concerning the ME response of such metallic glass/piezopolymer/metallic glass, being metallic glass = Vitrovac 4040 or home-made amorphous ribbons, and piezopolymer = PVDF or the 2,6(*β*-CN) polyimide or other copolyimides [15,56].

**Figure 12:**
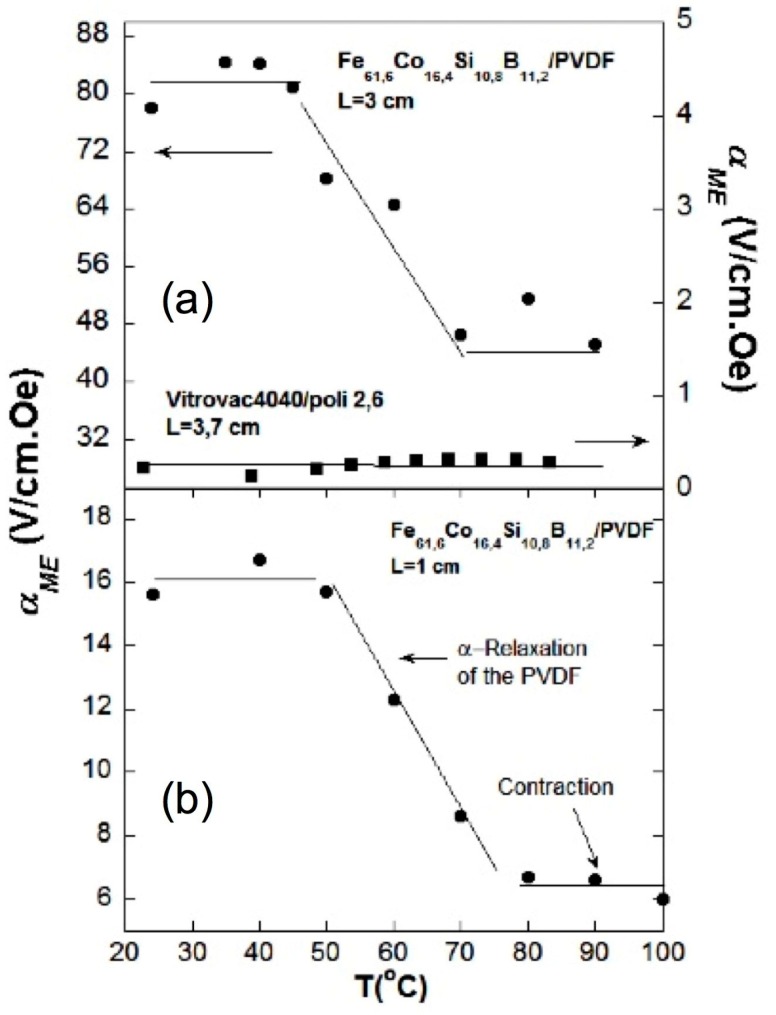
(**a**) Dependence of the magnetoelectric coefficient with temperature for (a) L = 3 cm Fe_61.6_Co_16.4_Si_10.8_B_11.2_/PVDF and L = 3.7 cm Vitrovac^®^ 4040/poli 2,6 laminated L-T composites; (**b**) L = 1 cm Fe_61.6_Co_16.4_Si_10.8_B_11.2_/PVDF laminated composite. All the measurements were carried out at the magnetoelastic resonance frequency of the laminates. Reprinted with permission of the authors from [15]. Copyright 2015 IEEE.

Figure 12 clearly shows that the behavior of the laminates fabricated with PVDF agrees with the temperature dependence of the PVDF polarization value. This fact also tell us about the optimized bonding conditions followed when fabricating the laminates [14,15], as previously mentioned in the Experimental section. The change that the measured magnetoelectric coefficient undergoes goes from 82 V/cm·Oe at room temperature down to 47 V/cm·Oe at 100 °C for the 3 cm long laminate. If we compare this behavior with the laminate fabricated with the high temperature poly 2,6 polyimide, it can be seen that despite the obtained value is much smaller (about 0.5 V/cm·Oe), the ME coefficient keeps constant up to 90 °C, which confirms the great stability that these high temperature poly and copolymides shows at high temperatures. Clearly, a future scope within this line of research hints for the synthesis of high temperature piezoelectric polymers with high value of the remnant polarization (or at least as high as the one of PVDF).

## 6. Conclusions/Outlook

Metallic glass/PVDF magnetoelectric (ME) laminated heterostructures show to date the highest values achieved for the induced ME voltage (over 300 V/cm·Oe) despite both constituents, piezopolymeric and magnetic, have not the best piezoelectric and magnetostrictive performance among their respective class of materials. This is due to the fact that the key parameter turns out to be the piezomagnetic value, that in the case of metallic glasses is among the highest ones, and the good bonding between magnetostrictive and piezoelectric constituents. Another factor is that the relative size of both constituents has been analyzed, giving as conclusion that the best performance is achieved when both components are of equal size. Nevertheless, reduction in the induced ME voltage as the laminated composite becomes shorter has been quantified, and losses of over 87% have been determined for laminates of 3 cm length and below.

When acting as magnetic field sensors, we have shown that the developed ME laminates work with a sensitivity of 1.5 V/Oe, a 70 nT resolution and averaged total drift of 0.04 Oe. Such performance can favorably compete with a reference Hall sensor used for comparison. On the other hand, the good capability of the reported ME devices to work as energy harvesters has been proved. It was demonstrated a stored power density value for a 3 cm length ME laminate of about 1.2 mW/cm^3^. This (magnetoelectric) power generated value is comparable to previously reported power densities for similar size laminates containing PZT as piezoelectric constituent (with much better piezoelectric performance that PVDF).

Finally, future tendencies point towards the development of new high temperature piezoelectric polymers for applications as directly monitoring of working machinery at the industry or aggressive environments (i.e., the desert, a tunnel or fighting a fire). Polyimides and more specifically, co-polymers of the type diamine 2CN (with good piezoelectric properties) + diamine 0CN (with good mechanical properties) have been tested as a good alternative to PVDF. Despite the modest value of the ME induced voltage measured, this new class of piezoelectric polymers shows an stable behaviour against temperature that makes them promising constituents of ME laminates in future high temperature applications.

## Figures and Tables

**Table 1:** Selected magnetic properties of the magnetostrictive sample compositions used in the fabrication of all ME laminates appearing in this work.

	μ_o_M_s_ (T)	χ	λ (ppm)	$d_{33}^{m}=\delta \lambda /\delta H$ (ppm/Oe)
	**(Fe_0.79_Co_0.21_)_75 + x_Si_15 − 1.4x_B_10 + 0.4x_**			
X = 0	1.3	55,000	18	1.4
X = 3	1.4	36,000	20	1.5
X = 6	1.7	50,000	23	2
	**Fe_85 − x_Co_x_B_15_**			
X = 21	1.9	70,000	25	2.8
	Vitrovac 4040 (Fe_39_Ni_39_Mo_4_Si_6_B_12_) *			
	0.8	>1000	8	1.4

* Values taken from Ref. [12].

**Table 2:** Comparison of some power output and power densities values reported so far for some ME laminates. *f_r_*, *H_ac_* and H* refer to the resonance (or working) frequency, the AC excitation field and DC field, respectively, for the maximum ME coupling. Table adapted from [23] with permission of the authors.

*f_r_* (kHz)	*H_ac_*(Oe)	H*(Oe)	P (µW)	P/V (mW/cm^3^)	Materials Involved
46.8	0.45	4.7	6.4	1.5 [23]	Fe_64_Co_17_Si_6.6_B_12.4_ and PVDF
66.1	1	66.1	0.065	0.7 [30]	Fe_0.7_Ga_0.3_ and PZT
26.9	0.3	50	917.7	0.956 [31]	FeNi and PZT
27.0	1	800	20	0.12 [26]	Terfenol-D and PZT
			93.6	0.1579 [32]	PVDF and PZT
			75,000	0.0051 [33]	APC 855 (piezoelectric)

**Table 3:** Value of the figure of merit of each used magnetostrictive ribbon (L = 3 cm) composition, in as quenched state.

	(Fe_0.79_Co_0.21_)_75 + x_Si_15 − 1.4x_B_10 + 0.4x_			Fe_85 − x_Co_x_B_15_
X	0	3	6	21
*FM*	0.99	0.72	1.15	1.75
